# Intracellular Phage Tail-Like Nanostructures Affect Susceptibility of Streptomyces lividans to Osmotic Stress

**DOI:** 10.1128/msphere.00114-23

**Published:** 2023-04-11

**Authors:** Toshiki Nagakubo, Shumpei Asamizu, Tatsuya Yamamoto, Manami Kato, Tatsuya Nishiyama, Masanori Toyofuku, Nobuhiko Nomura, Hiroyasu Onaka

**Affiliations:** a Graduate School of Agricultural and Life Sciences, Department of Biotechnology, The University of Tokyo, Tokyo, Japan; b Collaborative Research Institute for Innovative Microbiology (CRIIM), The University of Tokyo, Tokyo, Japan; c Faculty of Life and Environmental Sciences, University of Tsukuba, Tsukuba, Japan; d Life Science Research Center, College of Bioresource Sciences, Nihon University, Tokyo, Japan; e Microbiology Research Center for Sustainability (MiCS), University of Tsukuba, Tsukuba, Japan; The University of Iowa

**Keywords:** phage tail-like nanostructures, *Streptomyces*, osmotic stress

## Abstract

Contractile injection systems (CISs) are a large group of phage tail-like nanostructures conserved among bacteria. Despite their wide distribution, the biological significance of CISs in bacteria remains largely unclear except for a few unicellular bacteria. Here, we show that Streptomyces lividans—a model organism of filamentous Gram-positive bacteria with highly conserved CIS-related gene clusters—produces intracellular CIS-like nanostructures (*Streptomyces* phage tail-like particles [SLPs]) that affect phenotypes of this bacterium under hyperosmotic conditions. In contrast to typical CISs released from the cells, SLPs are localized in the cytoplasm of S. lividans. In addition, loss of SLPs leads to (i) delayed erection of aerial mycelia on hyperosmotic solid medium and (ii) decreased growth during the transition from exponential growth phase to stationary phase in hyperosmotic liquid medium. Localization of fluorescent protein-tagged SLPs showed partial correlation with cell wall synthesis-related proteins, including MreB, an actin-like cytoskeleton protein. Our pulldown assay and subsequent quantitative proteome analysis also suggest that 30S ribosomal proteins and cell wall-related proteins, including MreB, are coeluted with SLPs. Furthermore, an interaction assay using the recombinant proteins revealed a direct interaction between a sheath protein of SLP and ribosomal protein S16. Results of cross-linking experiments show indirect interactions between SLPs and translation elongation factors. These findings collectively suggest that SLPs are directly or indirectly associated with a protein interaction network within the cytoplasm of S. lividans and that SLP loss ultimately affects the susceptibility of the bacterium to certain stress conditions.

**IMPORTANCE** Recent bioinformatic analyses have revealed that CIS-related gene clusters are highly conserved in Gram-positive actinomycetes, especially members of the genus *Streptomyces* known for their ability to produce therapeutic antibiotics. While typical CISs are released from the cells and can act as protein translocation systems that inject effector proteins into the target cells, our results indicate the unique intracellular localization of SLPs, CIS-related nanostructures produced by S. lividans. In addition, the direct and indirect interactions of SLPs with cytoplasmic proteins and SLP localization within specific regions of mycelia suggest that the biological significance of SLPs is related to intracellular processes. Further, SLP loss leads to increased susceptibility of S. lividans to osmotic stress, suggesting that production of these phage tail-like nanostructures ultimately affects the fitness of the bacterium under certain stress conditions. This work will provide new insight into the phage tail-like nanostructures highly conserved in *Streptomyces* species.

## INTRODUCTION

Diverse classes of bacteria produce phage tail-like nanostructures with versatile functionalities. For example, Gram-negative bacterial type VI secretion systems (T6SSs) are cell envelope-spanning nanomachines which inject various effector proteins into target cells in a cell-cell contact-dependent manner, thereby playing an important role in interbacterial competition (1). Contractile injection systems (CISs) encompass another group of phage tail-like nanomachines that are widely distributed among Gram-negative bacteria, Gram-positive bacteria, and archaea (2, 3). Gram-negative bacterial CISs studied to date are released into the extracellular milieu and act as protein translocation systems by injecting effector proteins into eukaryotic target cells (4–6). *Serratia* species produce an antifeeding prophage (Afp) that exhibits killing activity toward a grass grub by injecting toxic effector proteins into the target cells (4). Another example of a CIS with a unique function is the metamorphosis-associated contractile structure (MAC) produced by Pseudoalteromonas luteoviolacea. In contrast to Afp, the MAC induces metamorphosis in marine tube worms, which is believed to be beneficial for target eukaryotes (6). Although these findings have illustrated the broad existence and intriguing biological functions of these phage-related nanomachines, current knowledge of the biological roles of CISs is quite limited for bacteria, with the exception of gammaproteobacteria.

The genus *Streptomyces*, which comprises filamentous Gram-positive bacteria, is now regarded as a major bacterial group in which CIS-related genes are highly conserved (3). These Gram-positive bacteria are ubiquitously found in natural environments, such as soil, and are known for their unique life cycle and ability to produce therapeutic antibiotic compounds. During their life cycle, they grow as substrate mycelia by apical tip extension after spore germination, followed by morphological differentiation from substrate mycelia to aerial mycelia triggered by several environmental factors, including nutritional changes (7). Aerial mycelia finally turn into spore chains. The production of CIS-related nanostructures, namely, *Streptomyces* phage tail-like particles (SLPs), by Streptomyces lividans and its close relative Streptomyces coelicolor A3(2) is regulated by *bldA*, a key regulatory gene involved in morphological differentiation and antibiotic production in *Streptomyces* (8–11). Although the deletion mutants of S. coelicolor and S. lividans for the CIS-related genes are phenotypically normal in terms of morphological differentiation and antibiotic production (10, 12), we previously reported that the loss of SLP genes affects microbial competition between S. lividans and fungal competitors, with the colonies of SLP-deficient mutants of S. lividans being more severely invaded by fungi than the parental strain (12). However, how SLPs contribute to the phenotype of S. lividans under competitive conditions remains unknown.

In *Streptomyces*, many genes have been identified as factors that mediate resistance to cell envelope stress, including osmotic stress (13). Among them, *sigE*, which encodes sigma factor E (SigE), is a key regulator of the maintenance of cell envelope integrity (14, 15). S. coelicolor mutants lacking *sigE* show impaired cell wall synthesis and are highly susceptible to a wide range of cell envelope stressors (14). Genome-wide analysis of the SigE regulon in S. coelicolor A3(2) revealed that the SigE regulon includes genes predicted to be involved in lateral cell wall synthesis in *Streptomyces* (15). Notably, *SCO4253* (a homologue of *slpS* that encodes a sheath protein) and *SCO4263* (a homologue of *slpR* that encodes a CIS-related gene cluster-specific transcriptional regulator) are also regulated by SigE (15).

In the present study, we show that the loss of SLPs in S. lividans TK23 affects the susceptibility of substrate mycelia to certain stress conditions that potentially lead to cell envelope stress. In contrast to known Gram-negative bacterial CISs released from cells, SLPs show intracellular localization in the cytoplasm of S. lividans mycelia. In addition, localization of fluorescent protein-tagged SLPs partially correlates with cell wall synthesis proteins during the late exponential growth phase. Additionally, we provide biochemical data implying an interaction involving SLPs, translation elongation factors, and ribosomal proteins.

## RESULTS

### SLPs are localized in the cytoplasm.

**FIG 1:**
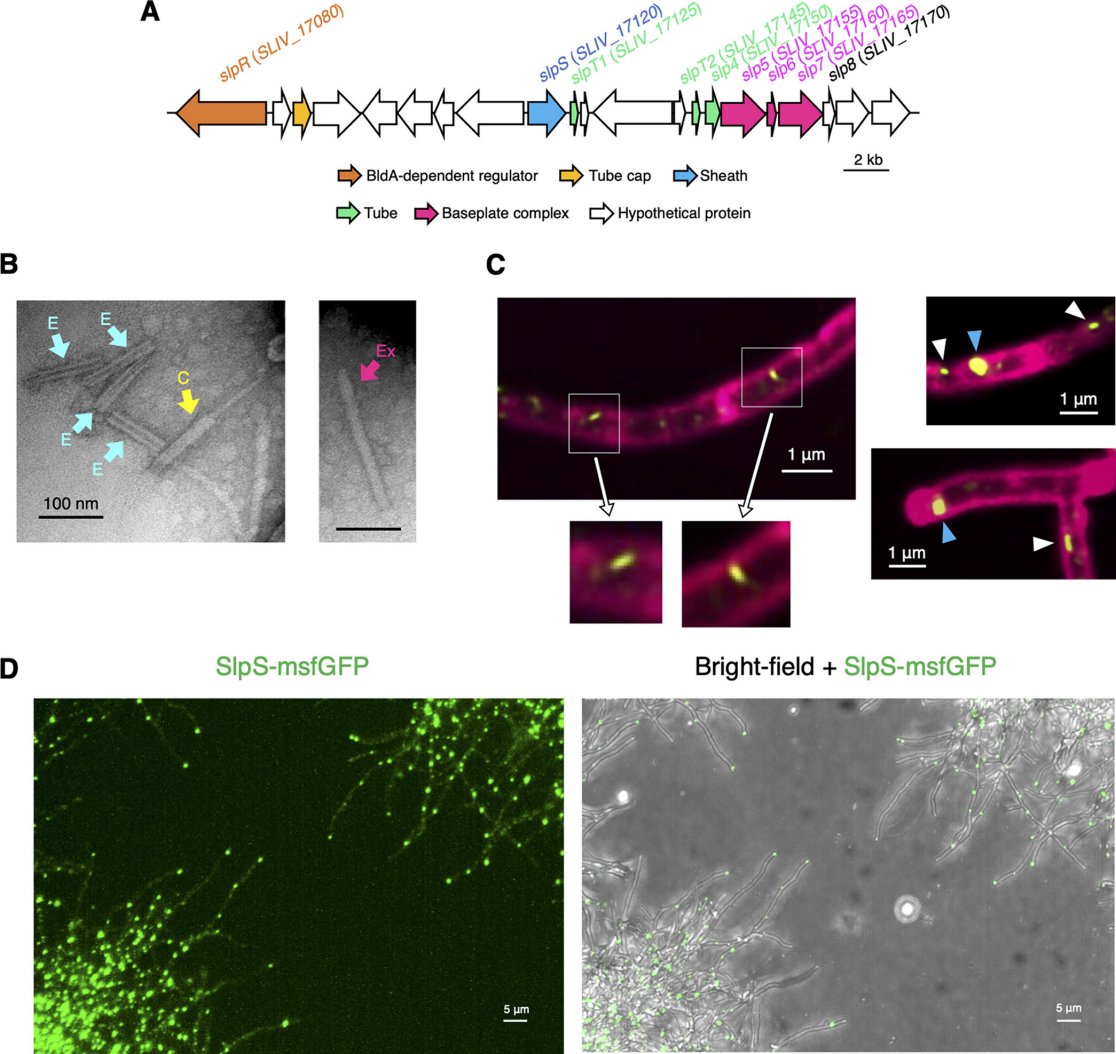
Localization of SLPs in growing S. lividans mycelia. SLPs show intracellular localization in the cytoplasm of S. lividans. (A) Organization of the SLP gene cluster. Colored arrows represent genes encoding SLP structural proteins and a cluster-specific regulator. White arrows represent hypothetical proteins with unknown function. Gene numbers correspond to the completed S. lividans genome in GenBank (accession no. CP009124.1). (B) TEM images of the extracted SLPs. Arrows indicate SLPs likely to be in each of the following states: Ex, extended state; C, contracted state; E, empty sheath. Scale bars, 100 nm. (C) The sheath protein (SlpS) was fused with msfGFP, and its localization was analyzed using CLSM. The strain expressing SlpS-msfGFP was constructed by replacing the *slpS* gene with the fusion gene at the native chromosomal locus. GFP-tagged SLPs are shown in yellow. The cell membrane was stained with FM4-64 dye (magenta). Microscopy settings for fluorescence imaging were as follows: microscope, Olympus SpinSR10 equipped with UAPON OTIRF 100 × 1.49 NA; excitation laser wavelength, 488 nm; laser intensity, 20%; exposure time, 500 ms; emission bandpass filter, 525/50 nm (SlpS-msfGFP) and 684/40 nm (FM4-64). The estimated length (100 to 200 nm) of the visualized SLP (left panel) is consistent with the result of TEM analysis in panel B. White and blue arrowheads in the right panels indicate smaller and larger foci of SlpS-msfGFP, respectively. Scale bars, 1 μm. (D) Microscopy analysis was conducted for a microcolony of S. lividans expressing SlpS-msfGFP. Left, GFP fluorescence; right, a merged image of GFP fluorescence and a bright-field image. Microscopy settings for the fluorescence imaging were as follows: microscope, Olympus SpinSR10 equipped with UPLAPO OHR 60 × 1.5 NA; excitation laser wavelength, 488 nm; laser intensity, 20%; exposure time, 500 ms; emission bandpass filter, 525/50 nm. Microscopy settings for the bright-field imaging were as follows: lamp intensity, 150; exposure time, 200 ms. Scale bars, 5 μm. Result of live cell imaging are shown in Movie S1 in the supplemental material. Results of a control experiment are shown in Fig. S2 at https://doi.org/10.6084/m9.figshare.22223797. Details are described in Materials and Methods.

The SLP gene cluster includes several genes encoding CIS-related proteins that would constitute the sheath, tube, and baseplate complex (Fig. 1A) (4–6). Transmission electron microscopy (TEM) images of the extracted SLPs showed the following three differential states that resemble those described in previous CIS studies: extended state, contracted state, and empty sheath (Fig. 1B) (5, 6, 16, 17). These states can be considered to represent the typical conformational transition of CISs during their action. The action mechanism encompasses tube ejection from the sheath-tube complex upon sheath contraction of a CIS particle in the extended state (Fig. 1B) (17). However, the SLP gene cluster lacks the viral genes encoding lytic proteins (Fig. 1A), which may potentially mediate cell lysis and ultimately lead to the release of CIS from the cells. This raised the possibility that SLP localization is different from that of the known CISs that are released from the producer cells and directly attach to the target cells (17). In a previous study, a Gram-negative bacterial extracellular CIS (eCIS)-related nanostructure that shows cell membrane-associated subcellular localization was discovered (18). Although this nanostructure is annotated as a subtype of T6SS (T6SS*^iv^*) because of its localization, the amino acid sequences of its components are closely related to CISs rather than T6SSs (18). Notably, the amino acid sequences of the SLP component are phylogenetically closely related to T6SS*^iv^* rather than functionally characterized Gram-negative bacterial CISs (see Fig. S1 at https://doi.org/10.6084/m9.figshare.22223797), and the SLP gene cluster and T6SS*^iv^* gene cluster are similar in terms of the lack of viral lytic systems (Fig. 1A) (18). Therefore, we wondered whether SLPs are localized in the cytoplasm of S. lividans. To test this hypothesis, we investigated SLP localization in S. lividans cultures by microscopic and immunological analyses. Since a previous study demonstrated that the release of CISs from the producer cells can be visualized using green fluorescent protein (GFP)-tagged CIS particles (6), we cultured an S. lividans strain expressing SlpS, a sheath protein of SLPs (Fig. 1A), fused with monomeric superfolder green fluorescent protein (msfGFP) (12, 19–21). This strain was constructed by replacing *slpS* with *slpS-msfgfp* at the native chromosomal locus and did not display any apparent defect in mycelial growth or morphology. Confocal laser scanning microscopy (CLSM) analyses showed that SLPs would be retained within the cytoplasm during the growth of S. lividans mycelia (Fig. 1C and D; see Movie S1 in the supplemental material; see also Fig. S2 and S3 at https://doi.org/10.6084/m9.figshare.22223797). Two different sizes of SlpS-msfGFP foci were observed, suggesting that the larger fluorescence foci represent groups of multiple SLPs at certain regions of mycelia (Fig. 1C). Moreover, we prepared antiserum specific for either SlpS or Slp5 (a homologue of the VgrG spike protein) and performed Western blot analyses to confirm the SLP localization. In our experiments, we could not detect SlpS in the culture supernatant isolated from liquid culture of S. lividans (see Fig. S4 at https://doi.org/10.6084/m9.figshare.22223797). We also isolated the cellular membrane from S. lividans mycelia by sonication and density gradient ultracentrifugation. Distribution of SLP proteins and the isolated cellular membranes throughout the fractions showed different patterns, suggesting that SLPs lack a tight association with cellular membranes (see Fig. S5 at https://doi.org/10.6084/m9.figshare.22223797).

10.1128/msphere.00114-23.1 MOVIE S1 Time-lapse imaging of SLP expression. This movie is related to Fig. 1. S. lividans strain expressing SlpS-msfGFP was grown on BeG medium and observed by CLSM. S. lividans was precultured on cellophane for 2 days and then subjected to CLSM analysis. Details of this experiment are described in Materials and Methods. Mycelia were cultivated for 38 h, and images were taken every hour. Bright-field images and msfGFP fluorescence (green) were merged. Scale bar, 30 μm. Download Movie S1, AVI file, 7.8 MB. Copyright © 2023 Nagakubo et al. 2023 Nagakubo et al. https://creativecommons.org/licenses/by/4.0/ This content is distributed under the terms of the Creative Commons Attribution 4.0 International license.

### Loss of SLPs leads to increased susceptibility to hyperosmotic stress.

**FIG 2:**
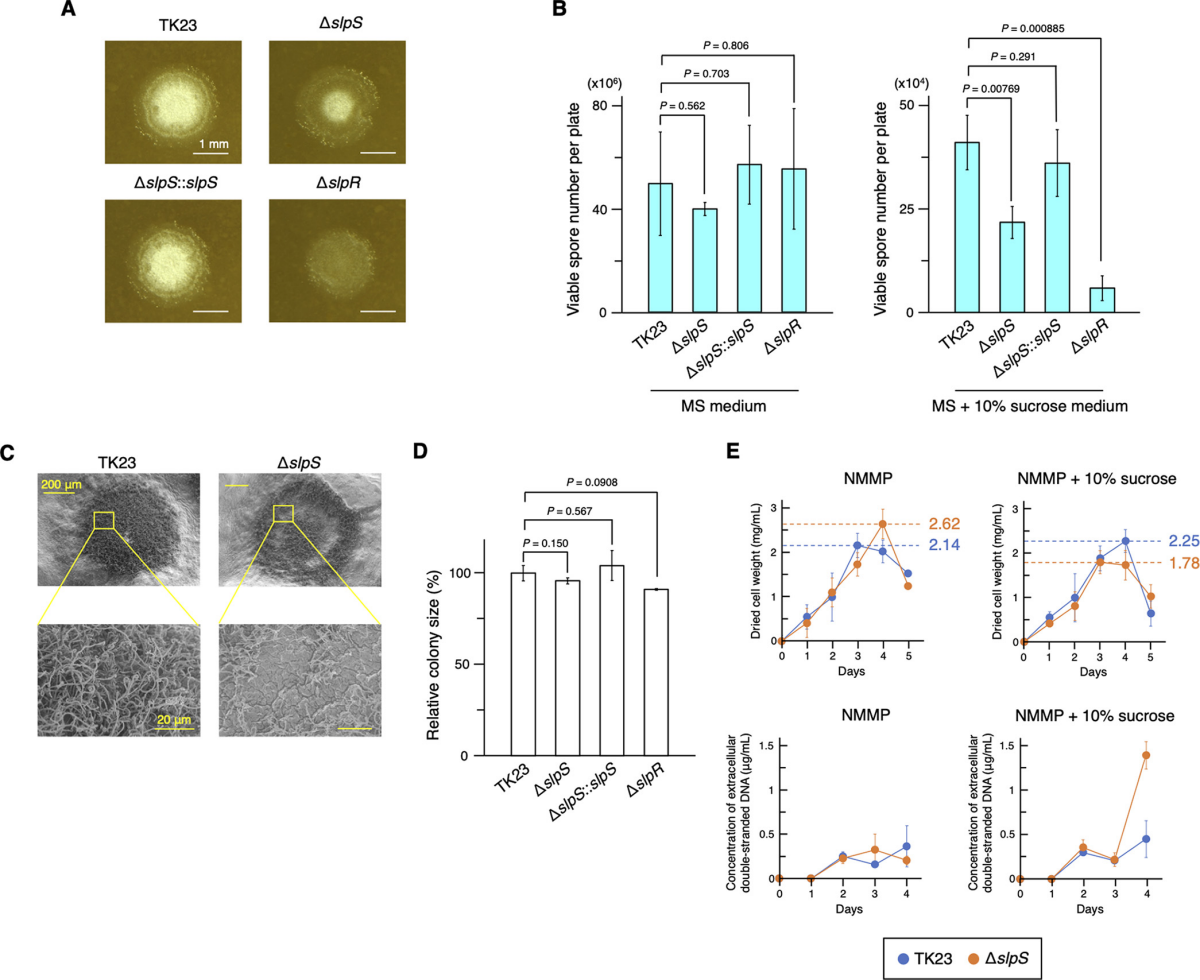
SLP loss affects susceptibility of S. lividans to hyperosmotic stress. Loss of SLP leads to increased susceptibility to nonionic osmotic stress. (A) SLP-deficient strains exhibit aberrant colony morphologies under high-osmolarity conditions. TK23 (parental strain), Δ*slpS* mutant, Δ*slpR* mutant, and *slpS*-complemented strains were grown on solid MS medium supplemented with 10% (wt/vol) sucrose for 4 days. The colony number per plate was adjusted to approximately 100. Scale bars, 1 mm. Related data are shown in Movies S2 and S3 in the supplemental material and in Fig. S7 at https://doi.org/10.6084/m9.figshare.22223797. (B) The S. lividans strains were grown on MS medium with or without sucrose supplementation (10%, wt/vol). After 4 days of incubation at 30°C, spores were isolated from the cultures and then spread onto fresh solid medium to count the number of viable spores. For spore formation on MS medium, colony numbers were adjusted to approximately 50 per plate. Bars represent mean values ± standard deviations (SD) for the spores isolated from three independent cultures. *P* values were calculated by *t* test with Welch’s correction. (C) SEM images of S. lividans strains grown on sucrose-supplemented MS medium. The parental strain and Δ*slpS* mutant of S. lividans show different colony morphologies. These strains were grown on MS medium supplemented with 10% (wt/vol) sucrose and subjected to SEM analysis as described in Materials and Methods. Scale bars, 200 μm (upper panels) and 20 μm (lower panels). (D) The S. lividans strains were grown on sucrose-supplemented MS medium for 4 days, and then the colony sizes were measured. The colony number was adjusted to approximately 100 per plate. Bars represent mean values ± SD for three independent cultures (total, 300 colonies for each strain). *P* values were calculated by *t* test with Welch’s correction. (E) The S. lividans strains were grown in liquid NMMP medium with or without 10% (wt/vol) sucrose supplementation. In this experiment, NMMP liquid medium was used to obtain dispersed growth of S. lividans and to measure the extracellular DNA concentration precisely. All values represent mean values ± SD for three independent cultures. Dashed lines indicate the maximum mean values of the dry cell weights. The concentration of extracellular double-stranded DNA at 5 days is not shown because the values at this time point were highly variable, probably due to the presence of cellular materials released by frequent cell lysis, which may have interfered with the measurement. Extracellular DNA concentration was quantified using a PicoGreen assay, as described in Materials and Methods.

Given the intracellular localization of SLPs, we were interested in the effects of their loss on the phenotypes of S. lividans. SigE-dependent regulation of SLP genes (*slpS* and *slpR* homologues) has been reported in S. coelicolor A3(2) (15). In the present study, we confirmed that SlpS expression is also dependent on SigE in S. lividans as well as in S. coelicolor A3(2) at the protein level (see Fig. S6 at https://doi.org/10.6084/m9.figshare.22223797). Inspired by these observations, we examined the effects of SLP loss on the phenotypes of S. lividans under growth conditions that promote cell envelope stress. We found that the SLP-deficient strains and the parental strain showed different phenotypes under high-osmolarity conditions. The S. lividans strain lacking *slpS* showed a colony morphology with delayed aerial mycelial erection on mannitol soy flour (MS) medium supplemented with 10% sucrose to create a high-osmolarity environment in the medium (Fig. 2A; see Movies S2 and S3 in the supplemental material; see also Fig. S7 at https://doi.org/10.6084/m9.figshare.22223797). This aberrant phenotype of the Δ*slpS* mutant grown on the sucrose-supplemented medium was observed in ~97% of the colonies and could be restored by *slpS* complementation (Fig. 2A). Delayed aerial mycelial erection in the Δ*slpR* mutant, which lacks the SLP gene cluster-specific regulator SlpR (Fig. 2A) (9, 10, 12), was observed in ~100% of the colonies, further confirming the involvement of SLPs in the phenotypic changes (Fig. 2A). Additionally, we cultivated these strains on MS medium with or without sucrose supplementation and then isolated spores from the cultures to compare spore formation rates. In this experiment, SLP-deficient strains showed remarkably lower spore formation rates than the parental strain and *slpS*-complemented strain in the sucrose-supplemented medium, while there was no significant difference between the strains on normal MS medium without sucrose supplementation (Fig. 2B). These observations are supported by a previous study reporting morphological defects in transposon mutants of S. coelicolor A3(2) for CIS-related genes (*slpS* homologue [*SCO4253*] or the *slpT2* homologue [*SCO4248*]) on the high-osmolarity MS medium (13). Scanning electron microscopy (SEM) analysis revealed that aerial mycelia were rarely observed in the midperipheral region of a colony of the Δ*slpS* mutant on the sucrose-supplemented medium (Fig. 2C). The similar colony sizes of strain TK23 and the Δ*slpS* mutant under the same growth conditions indicate that mycelial extension during vegetative growth was not severely affected by the absence of SlpS (Fig. 2A to D; see Fig. S7 at https://doi.org/10.6084/m9.figshare.22223797). Replacement of *slpS* with *slpS-msfgfp* at the native chromosomal locus did not lead to apparent growth defects on MS plus 10% sucrose medium, indicating that the C-terminal fusion of msfGFP did not affect the involvement of SLPs with the phenotype under nonionic osmotic stress conditions (see Fig. S8 at https://doi.org/10.6084/m9.figshare.22223797). Given that sucrose supplementation to the medium seemed to affect only aerial mycelial formation of SLP-deficient mutant colonies (Fig. 2A to D; see Fig. S7 at https://doi.org/10.6084/m9.figshare.22223797), these results suggest that loss of SLPs may lead to an increased sensitivity of the mycelia to nonionic osmotic stress during the transition into aerial mycelia rather than a decrease in vegetative growth rate. Thus, we assessed the effects of high-osmolarity conditions on the growth of the Δ*slpS* mutant and the parental strain of S. lividans in liquid medium, in which S. lividans does not undergo a morphological transition from substrate mycelia to aerial mycelia (22). These strains were cultivated in a synthetic NMMP liquid medium (see “Strains and culture conditions” below) with or without 10% (wt/vol) sucrose supplementation. In normal NMMP medium, the Δ*slpS* mutation led to a slight increase in cell weight. On the other hand, in high-osmolarity NMMP medium, the maximum value of the cell weight was significantly lower than that in normal NMMP medium (day 4 in NMMP versus day 3 in NMMP plus sucrose; *P = *0.0204, *t* test with Welch’s correction), whereas the growth of the parental strain was apparently unaffected (Fig. 2E; see Fig. S9 at https://doi.org/10.6084/m9.figshare.22223797). Furthermore, the normalized concentration of extracellular double-stranded DNA during 3 to 4 days of cultivation rapidly increased in the Δ*slpS* mutant exposed to high-osmolarity conditions (day 4, TK23 versus Δ*slpS* mutant; *P = *0.0757, *t* test with Welch’s correction), indicating that the increased cell lysis during this period may account for the decrease in cell weight (Fig. 2E). Given that the growth rates of the parental strain and the Δ*slpS* strain during early to mid-exponential growth phase were almost identical, these results suggest that the deletion of *slpS* affects the susceptibility of S. lividans to nonionic osmotic stress when the cultures are transitioning into the stationary phase. Furthermore, the addition of 0.5 M NaCl to MS medium also led to a decrease in spore formation rates in the SLP-deficient mutants (see Fig. S10 at https://doi.org/10.6084/m9.figshare.22223797).

10.1128/msphere.00114-23.2 MOVIE S2 Parental strain of S. lividans grown under high-osmolarity conditions. This movie is related to Fig. 2. The parental strain (TK23) of S. lividans was grown on MS medium supplemented with 10% (wt/vol) sucrose. S. lividans was precultured on the medium for 2 days and then subjected to microscopic analysis. The spore number was adjusted to approximately 100 per plate. Scale bar, 2,000 μm. Download Movie S2, AVI file, 12.0 MB. Copyright © 2023 Nagakubo et al. 2023 Nagakubo et al. https://creativecommons.org/licenses/by/4.0/ This content is distributed under the terms of the Creative Commons Attribution 4.0 International license.

10.1128/msphere.00114-23.3 MOVIE S3 Δ*slpS* mutant of S. lividans grown under high-osmolarity conditions. This movie is related to Fig. 2. The Δ*slpS* mutant of S. lividans was grown on MS medium supplemented with 10% (wt/vol) sucrose. S. lividans was precultured on the medium for 2 days and then subjected to microscopic analysis. The spore number was adjusted to approximately 100 per plate. Scale bar, 2,000 μm. Download Movie S3, AVI file, 11.0 MB. Copyright © 2023 Nagakubo et al. 2023 Nagakubo et al. https://creativecommons.org/licenses/by/4.0/ This content is distributed under the terms of the Creative Commons Attribution 4.0 International license.

### SLP localization is partially associated with cell wall synthesis.

Impaired growth under hyperosmotic conditions has been previously reported in *Streptomyces* mutants lacking cell envelope-related genes, such as *mreB*, which encodes an actin-like protein, MreB, and is regulated by SigE (see Fig. S6 at https://doi.org/10.6084/m9.figshare.22223797) (15, 23, 24). MreB is widely conserved among bacteria, including Escherichia coli, Bacillus subtilis, and *Streptomyces* species (23–25). In many unicellular bacteria, MreB plays a central role in lateral cell wall synthesis by interacting with cell wall synthesis proteins and supporting the proper localization of the cell wall synthesis machinery (26, 27). Owing to this important role of MreB in cell wall synthesis, MreB malfunction severely impacts cell envelope integrity, leading to loss of cell shape, increased sensitivity to cell envelope stress, and decreased growth in liquid medium (28, 29). Although *Streptomyces* MreB is not a determinant of cell shape (see Fig. S11 at https://doi.org/10.6084/m9.figshare.22223797) (23, 24), it is consistently expressed and forms a cell wall-synthesizing complex with other Mre proteins, including MreC, MreD, PBP2, FtsI, and RodZ, that closely resembles the lateral wall-synthesizing complex produced by rod-shaped bacteria (24, 30). Importantly, mycelia of the Δ*mreB* mutant of S. coelicolor are highly sensitive to osmotic stress, similar to the Δ*sigE* mutant with impaired cell envelope integrity (23, 24). In the present study, we confirmed that this characteristic of the Δ*mreB* mutant of S. coelicolor A3(2) was also observed in the Δ*mreB* mutant of S. lividans (see Fig. S12 at https://doi.org/10.6084/m9.figshare.22223797). We also found that the maximum cell weight of the Δ*mreB* mutant of S. lividans in liquid medium was markedly decreased (see Fig. S13 at https://doi.org/10.6084/m9.figshare.22223797), similar to the reduced growth of unicellular bacteria deficient in MreB filaments (29).

**FIG 3:**
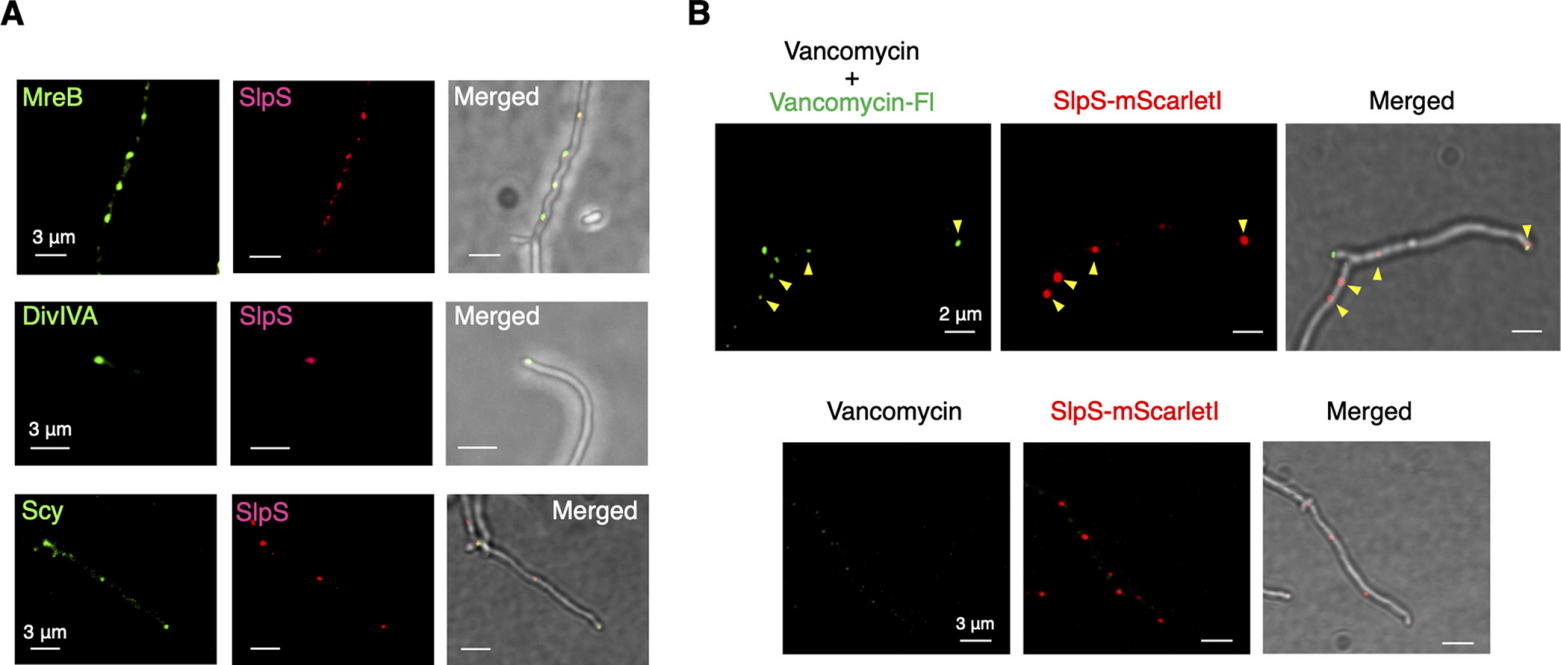
Partial correlation of SLP localization with cell wall synthesis in S. lividans. (A) Representative images of colocalization analysis. Left, msfGFP fluorescence. Middle, mScarletI fluorescence. Right, merged image of msfGFP fluorescence, mScarletI fluorescence, and bright-field image. Each protein was fused with the fluorescent protein as described in Materials and Methods. Scale bars, 3 μm. A nonmembrane control is shown in Fig. S2B at https://doi.org/10.6084/m9.figshare.22223797. (B) Cell wall synthesis was visualized using a vancomycin-Fl conjugate (Van-Fl). S. lividans substrate mycelia were grown in a BeG slide culture for 3 days and then stained with 1 μM Van-Fl. To minimize nonspecific staining by Van-Fl, vancomycin without a fluorophore (2 μM) was also added. The stained mycelia were observed within 3 min after the treatment. Yellow arrowheads indicate regions in which SlpS-mScarletI foci overlap Van-Fl foci. Scale bars, 2 or 3 μm. Microscopy settings for the fluorescence imaging were as follows: microscope, Olympus SpinSR10 equipped with UAPON OTIRF 100 × 1.49 NA; excitation laser wavelength, 488 nm (msfGFP fusion proteins and Van-Fl dye) and 561 nm (mScarletI); laser intensity, 30%; exposure time, 500 ms; emission bandpass filter, 525/50 nm (msfGFP fusion proteins and Van-Fl dye) and 617/73 nm (mScarletI fusion proteins). Microscopy settings for bright-field imaging were as follows: lamp intensity, 150; exposure time, 200 ms.

We constructed an S. lividans strain expressing MreB-msfGFP by replacing *mreB* with the fusion gene *mreB-msfgfp* at the native chromosomal locus. CLSM analysis revealed that MreB-msfGFP formed discrete foci in substrate mycelia (Fig. 3A). The expression of the GFP-labeled MreB under the regulation of the native promoters had no apparent effect on the growth of S. lividans (see Fig. S12 and S13 at https://doi.org/10.6084/m9.figshare.22223797), consistent with previous studies involving S. coelicolor A3(2) (23). To further confirm the formation of the MreB filaments in S. lividans, we treated the MreB-msfGFP-expressing S. lividans strain with the MreB filamentation inhibitor A22 (29). In the presence of A22, MreB-msfGFP fluorescence showed a dispersed distribution within the cytoplasm, indicating that MreB-msfGFP foci were derived from the polymerized MreB-msfGFP (see Fig. S14 at https://doi.org/10.6084/m9.figshare.22223797). Next, we analyzed the localization of MreB and SlpS in a strain expressing both MreB-msfGFP and SlpS-mScarletI under the regulation of their native promoters. This strain was constructed as described in Materials and Methods. Using CLSM, we found that 76.6% ± 13.6% of the SlpS-mScarletI foci overlapped MreB-msfGFP filaments during the late exponential growth phase (100 mycelia were analyzed; average Spearman’s correlation coefficient, 0.753; Costes’ statistical confidence, *P* > 95%) (Fig. 3A). We also performed colocalization analysis using DivIVA (31) and Scy (32), localizations of which are known to be associated with cell wall synthesis. We constructed S. lividans strains expressing SlpS-mScarletI and either DivIVA-msfGFP (31) or msfGFP-Scy (32) as described in Materials and Methods. In our observations, expression of these GFP-tagged proteins under regulation of their native promoters did not affect the apparent phenotypes of S. lividans during vegetative growth. Moreover, 63.9% ± 8.5% and 71.1% ± 10.3% of SlpS-mScarletI foci overlapped the foci of GFP-tagged DiviVA and Scy, respectively, within the substrate mycelia during the late exponential growth phase (100 mycelia were analyzed for each strain; average Spearman’s correlation coefficient, 0.409 for DivIVA and 0.401 for Scy; Costes’ statistical confidence, *P* > 95%) (Fig. 3A). While DivIVA and Scy are thought to control apical tip extension and mycelial branching (31, 32), tip extension velocities and branching frequencies of substrate mycelia were comparable between the parental strain and the Δ*slpS* strain of S. lividans (see Fig. S15 at https://doi.org/10.6084/m9.figshare.22223797), indicating that SLP, as well as MreB, is not involved in the regulation of mycelial extension and branching. In addition, we visualized the regions undergoing cell wall synthesis in S. lividans by using a vancomycin-Fl conjugate (Van-Fl), a dye that stains the Ala-Ala terminal moiety of the newly synthesized peptidoglycan. Van-Fl accumulation was observed at apical tips and specific regions of the lateral cell wall during the late stage of vegetative growth (Fig. 3B). In addition, 77.4% ± 12.8% of SlpS-mScarletI foci overlapped Van-Fl spots (100 mycelia were analyzed; average Spearman’s correlation coefficient, 0.381; Costes’ statistical confidence, *P* > 95%) (Fig. 3B). Van-Fl spots were rarely found in the lateral cell wall of the Δ*slpS* mutant (see Fig. S16A and B at https://doi.org/10.6084/m9.figshare.22223797). Furthermore, we incubated mycelia of S. lividans *slpS-mScarletI* with a synthetic d-amino acid with a fluorescent moiety (HADA, 3-[[(7-Hydroxy-2-oxo-2*H*-1-benzopyran-3-yl)carbonyl]amino]-D-alanine), a peptidoglycan-labeling dye (33). After a short period of incubation (30 min), we could detect partial HADA incorporation at sites of SlpS-mScarletI foci (see Fig. S16C at https://doi.org/10.6084/m9.figshare.22223797). Collectively, these results indicate that SLP localization is partially associated with cell wall synthesis in S. lividans mycelia at the late stage of vegetative growth phase.

### Interactions of SLPs with cytoplasmic proteins.

**FIG 4:**
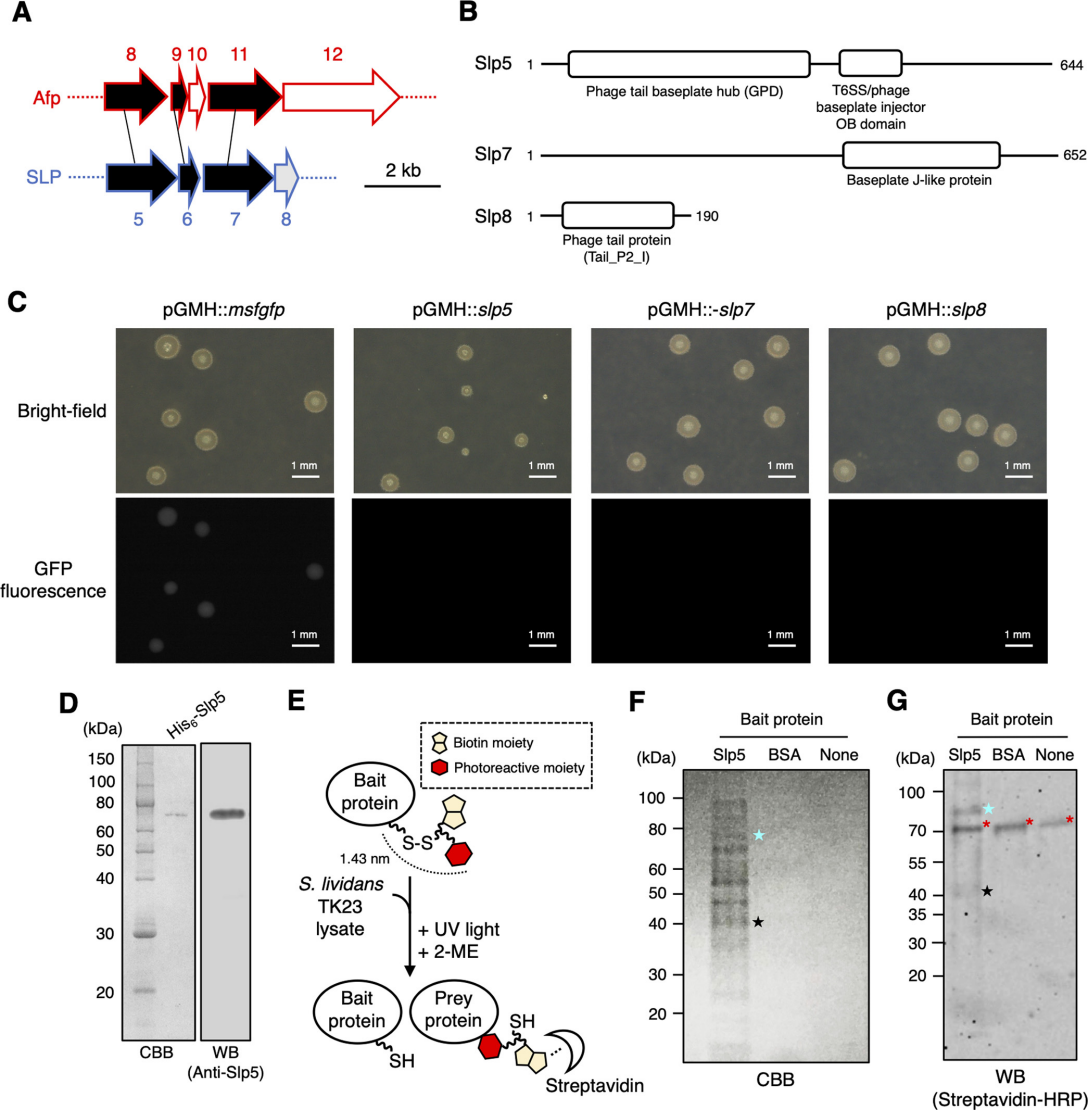
Indirect interactions between Slp5 and translation elongation factors inferred from a cross-linking experiment. Potential interaction partners of SLP were identified by biochemical analyses. (A) The organization of genes encoding baseplate complexes of SLP and that of Afp gene clusters were compared. Black lines and arrows indicate the genes sharing amino acid sequence homology with each other. A gray arrow indicates the gene sharing homology with other phage genes rather than Afp genes. (B) Organizations of Pfam domains of SLP baseplate-related proteins. (C) S. lividans strains overexpressing msfGFP or each of SLP proteins (Slp5, Slp7, and Slp8) were grown on solid BeG medium containing 10 μg/mL thiostrepton for 2 days. GFP fluorescence is shown in gray scale. Scale bars, 1 mm. Note that the colonies overexpressing Slp5 are significantly smaller than those of the other conditions. Colony numbers were adjusted to approximately 100 per plate. (D) Slp5 tagged with His_6_ at the N terminus was expressed in E. coli and purified. The purified protein was analyzed by SDS-PAGE. Details of the procedures are described in Materials and Methods. CBB, Coomassie brilliant blue. WB, Western blot. His_6_-Slp5 was detected by anti-Slp5 antiserum and HRP secondary antibody. (E) Schematic diagram of cross-linking using sulfo-SBED. Details of the procedure are described in Materials and Methods. Surface-exposed amino groups of the purified bait protein were initially labeled with the electrophilic cross-linking reagent, and then the interaction partner (prey protein) and the labeled bait proteins were cross-linked via photoactivation of the phenylazide group. The S-S bond was subsequently cleaved by 2-mercaptoethanol (2-ME). The resulting prey protein containing the biotin moiety was captured by a streptavidin column and then eluted. (F) Proteins captured by sulfo-SBED cross-linking were analyzed by SDS-PAGE. Black and blue stars indicate the bands corresponding to elongation factor Tu1 and elongation factor G1, respectively. (G) The proteins separated in panel F were further analyzed by Western blotting. Biotin-labeled proteins were detected using streptavidin-HRP conjugate. The proteins indicated by black and blue stars correspond to those shown in panel F. Red asterisks indicate the signals derived from an endogenous protein which would bind to streptavidin.

Given the SLP localization at certain regions of the cytoplasm, we examined the association of SLP with a protein-protein interaction network(s) within S. lividans mycelia. Bioinformatic analysis of the SLP gene cluster revealed the lack of potential interaction partners of several baseplate complex-related proteins. Specifically, in the SLP gene cluster, there is no candidate for a spike tip protein and an Afp12 homologue, which are potential interaction partners of Slp5 (probable VgrG-like spike protein; Afp8 homologue) and Slp7 (probable baseplate wedge protein; Afp11 homologue), respectively (Fig. 4A and B). Because homologues of Slp5 and Slp7 are surface exposed in the reported Afp structure (16), these proteins could be the interface between SLP and an unknown interaction partner(s). In addition, Slp8 is predicted to be a homologue of a phage tail-associated protein, which is possibly a member of the baseplate complex. We performed an overexpression experiment of these SLP proteins to identify a key protein(s) mediating the hypothetical interaction between SLPs and other cellular components. We constructed a series of plasmids harboring each of the *msfgfp*, *slp5*, *slp7*, and *slp8* genes under the regulation of an *hrdB* promoter that enables constitutive overexpression of each of these proteins during vegetative growth. Among these proteins, overexpression of Slp5 led to a significant delay in the growth of S. lividans (Fig. 4C). Since a domain with potentially toxic functions was not found in Slp5 sequences (Fig. 4B), this result suggests that the overexpression of Slp5 may perturb, by an uncontrolled protein-protein interaction, important cellular processes involved in the growth of S. lividans mycelia. We attempted to capture potential interaction partners of Slp5 or proteins located near Slp5 in the hypothetical protein complex by cross-linking and affinity-based pulldown assays. His-tagged Slp5 was heterologously expressed in E. coli and purified. The surface-exposed -NH_2_ groups of the purified Slp5 and bovine serum albumin (BSA) were labeled with sulfo-*N*-hydroxysuccinimidyl-2-(6-[biotinamido]-2-(*p*-azido benzamido)-hexanoamido) ethyl-1,3′-dithioproprionate (sulfo-SBED) (Fig. 4D and E). This trifunctional photoactivatable cross-linker with a relatively long spacer arm has been employed to identify nearest-neighbor proteins in complex macromolecular structures and organelles (34). Each of the labeled bait proteins (Slp5 and BSA) was incubated with cell lysates of S. lividans strain TK23, and biotin-labeled prey proteins were captured by a streptavidin column. While no visible band of prey protein appeared after SDS-PAGE and subsequent Coomassie brilliant blue (CBB) staining when BSA was used as a bait protein to detect nonspecific interaction, we could detect several bands by using Slp5 as a bait protein (Fig. 4F). Among these, two bands were found to be labeled by a biotin moiety (Fig. 4G). To identify these biotin-labeled prey proteins, we performed mass spectrometry analysis and found that these proteins were elongation factor Tu1 (EF-Tu1) and elongation factor G1 (EF-G1) encoded by the *tuf1* and *fusA* genes, respectively, of the S. lividans genome. EF-Tu, which contains a GTPase domain, delivers aminoacyl-tRNA to the A site of the ribosome and then dissociates upon hydrolysis of GTP (35, 36). EF-G also contains a GTPase domain, and its entire structure resembles the complex of EF-Tu and tRNA. EF-G catalyzes the movement of mRNA and tRNA with respect to the ribosome after the peptidyl transfer (37). The result of our mass spectrometry analysis suggested that biotin labeling occurred on a region of EF-Tu1 at amino acid positions 26 to 34 within the GTPase domain (also referred to as domain I) and a region of EF-G1 at amino acid positions 506 to 511 corresponding to domain III of typical EF-G in other bacterial species (see Fig. S17 at https://doi.org/10.6084/m9.figshare.22223797) (37).

**FIG 5:**
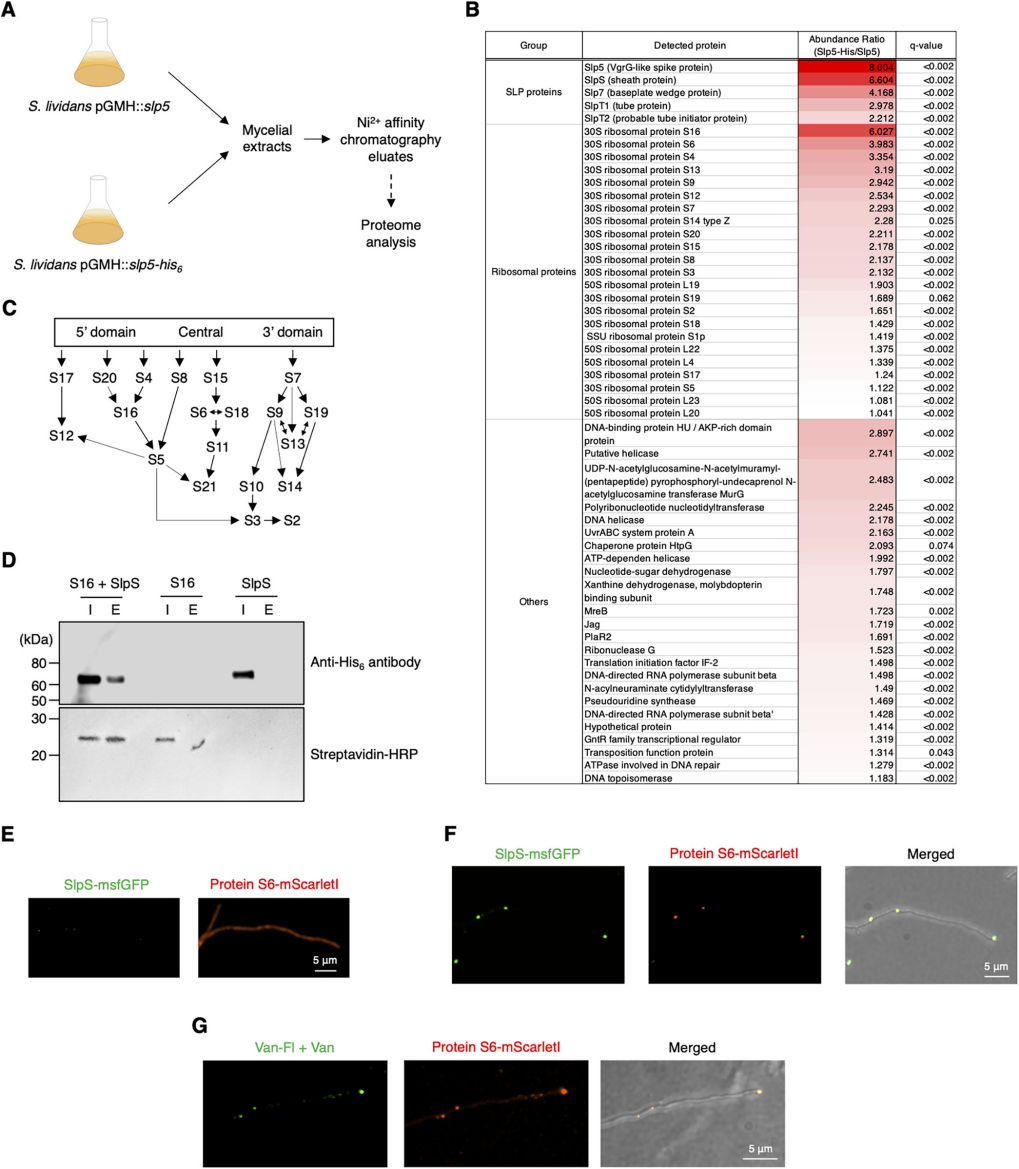
Interaction between SLP and ribosomal proteins. Quantitative proteome analysis and a coelution assay showed a possible interaction between SLP and 30S ribosomal proteins. (A) Scheme of the quantitative proteome analysis. S. lividans strains expressing either Slp5 or Slp5-His_6_ from pGMH plasmid were cultivated, and then the mycelial extracts at late exponential growth phase were subjected to Ni^2+^ affinity chromatography to enrich Slp5-His_6_. The resultant eluates were subjected to quantitative proteome analysis. Both strains were grown in three independent cultures. In total, six eluates were prepared and used for further liquid chromatography/mass spectrometry analysis. (B) List of proteins significantly enriched in Slp5-His_6_ eluates. The abundance ratio is the abundance of each of the detected proteins in the Slp5-His_6_ eluates relative to that of control Slp5 samples and is shown as a heat map. The data obtained from six independent cultures of Slp5-His_6_ and Slp5 strains (three cultures for each strain) were used in this analysis. (C) Putative assembly map of 30S ribosomal proteins. This assembly map is based on previous studies of the E. coli ribosome (39). (D) Results of the coelution assay of the recombinant ribosomal protein S16 and SlpS. Ni^2+^ affinity-purified StrepTagII-protein S16-His_10_ and SlpS-His_6_ were mixed at final concentrations of 0.2 and 0.1 mg/mL, respectively, and then subjected to coelution using a streptavidin column that selectively capture the tagged protein S16. I, input; E, eluate. Solutions containing either StrepTagII-protein S16-His_10_ or SlpS-His_6_ were used as controls. Detailed procedures are described in Materials and Methods. (E and F) Representative images of S. lividans *slpS-msfgfp rpsF-mscarletI* mycelia at the early (E) or late (F) stage of vegetative growth. Each protein was fused with the fluorescent protein as described in Materials and Methods. In panel F, a merged image of msfGFP fluorescence, mScarletI fluorescence, and bright-field image is also shown. Scale bars, 5 μm. (G) Cell wall synthesis was visualized using a vancomycin-Fl conjugate (Van-Fl). S. lividans *rpsF-mscarletI* mycelia at the late stage of vegetative growth were stained with 1 μM Van-Fl. To minimize nonspecific staining by Van-Fl, vancomycin without a fluorophore (2 μM) was also added. The stained mycelia were observed within 3 min after treatment. Scale bars, 5 μm. Microscopy settings for the fluorescence imaging were as follows: microscope, Olympus SpinSR10 equipped with UAPON OTIRF 100 × 1.49 NA; excitation laser wavelength, 488 nm (msfGFP fusion proteins and Van-Fl dye) and 561 nm (mScarletI fusion protein); laser intensity, 30%; exposure time, 500 ms; emission bandpass filter, 525/50 nm (msfGFP fusion proteins and Van-Fl dye) and 617/73 nm (mScarletI fusion protein). Microscopy settings for bright-field imaging were as follows: lamp intensity, 150; exposure time, 200 ms.

We also performed a pulldown assay and subsequent proteomic analysis to identify proteins associated with the partially purified SLP fraction (Fig. 5A). We constructed an S. lividans strain harboring pGMH::*slp5-His_6_*, and mycelia at mid- to late exponential growth phase were collected and then disrupted. The strain harboring pGMH::*slp5* was used as a control. It is important to note that this overexpression system for Slp5 and Slp5-His_6_ in S. lividans has been designed to express proteins at a moderate level to circumvent unwanted growth inhibition of the bacterium upon protein overexpression. Each of the mycelial extracts was subjected to Ni^2+^ affinity chromatography, and each of the eluates was then subjected to liquid chromatography-mass spectrometry analysis to identify proteins coeluted with Slp5-His_6_ (Fig. 5A). To rule out proteins detected via nonspecific interactions with the resin, we performed statistical analysis to identify proteins significantly enriched in the Slp5-His_6_ eluates. Major SLP structural proteins (SlpS [sheath], SlpT1 [tube], Slp4 [probable tube initiator], Slp7 [probable baseplate wedge protein], and Slp8 [probable baseplate protein]) were enriched in Slp5-His_6_ eluates compared with the tag-free controls, suggesting that Slp5-His_6_ was incorporated into SLPs and that the resulting His-tagged SLPs were isolated by the affinity chromatography (Fig. 5B). Notably, MurG, a cytoplasmic enzyme catalyzing a key step of peptidoglycan synthesis, and MreB were significantly enriched in the Slp5-His_6_ eluates (Fig. 5B). In addition, 17 of 21 proteins of the 30S ribosomal subunit were also enriched in the eluates (Fig. 5B). Overall, the proteins in the 5′ domain of 16S rRNA tended to be abundant in the Slp5-His_6_ eluates (Fig. 5B and C) (38). Since protein S16 showed the highest abundance ratio of 6.03, which is comparable to that of SlpS (6.60) (Fig. 5B), this protein could be an interaction partner of SLP proteins. In the S30 assembly map, S16 is next to protein S12, which contacts the tRNA bound to EF-Tu (36, 38, 39). To confirm the potential interaction between ribosomal proteins and SLP, we prepared the recombinant StrepTagII-protein S16-His_10_ and performed a coelution assay with the recombinant SLP proteins using a streptavidin column that can selectively capture the Strep-tagged protein. SlpS-His_6_ was detected in the eluate by Western blot analysis when the Strep-tagged ribosomal protein S16 was mixed with SlpS-His_6_ and the resultant mixture was subsequently loaded onto the column (Fig. 5D). SlpS-His_6_ was not detected in the eluate in the absence of the Strep-tagged protein S16 (Fig. 5D), suggesting a protein-protein interaction between SlpS and ribosomal protein S16 and providing an explanation for the high abundance ratio of ribosomal protein S16 in the quantitative proteome analysis (Fig. 5B). Although we also tested Slp5, Slp7, and Slp8 for the coelution assay, we failed to detect an interaction between the tagged ribosomal protein S16 and each of these SLP proteins. To further confirm the interaction between the 30S ribosomal subunit and SLP in S. lividans mycelia, we constructed a strain expressing ribosomal protein S6-mScarletI and SlpS-msfGFP under their native promoters. In our observation, expression of ribosomal protein S6-mScarletI had no apparent effect on the growth and mycelial morphology of S. lividans. Ribosomal protein S6-mScarletI fluorescence showed a uniform distribution within early vegetative mycelia in which the expression level of *slpS* was low (Fig. 5E; see Fig. S4 at https://doi.org/10.6084/m9.figshare.22223797). Discrete fluorescence foci of ribosomal protein S6-mScarletI were formed during the late stage of vegetative growth (Fig. 5F). Of these foci, 95.9% ± 3.6% overlapped SlpS-msfGFP foci, indicating that 30S ribosomal subunits and SLPs are significantly colocalized within S. lividans mycelia at the specific growth stage probably via the protein-protein interaction (100 mycelia were analyzed; average Spearman’s correlation coefficient, 0.634; Costes’ statistical confidence, *P* > 95%). Furthermore, we also constructed a strain expressing ribosomal protein S6-mScarletI and examined a correlation between 30S ribosomal subunit localization and cell wall synthesis. Van-Fl staining of mycelia of this strain revealed that 77.2% ± 12.1% of protein S6-mScarletI foci overlapped Van-Fl spots (Fig. 5G) (100 mycelia were analyzed; average Spearman’s correlation coefficient, 0.329; Costes’ statistical confidence, *P* > 95%). Collectively, these results suggest that the physical interaction of SLP with the 30S ribosomal subunit is partially associated with cell wall synthesis in S. lividans.

## DISCUSSION

Recent advances elucidating the structures and functions of phage tail-like nanostructures produced by microorganisms have revealed their structural similarity and functional versatility. Generally, the mechanistic models of known CISs have been built based on their homology to those of contractile phages (17, 40). Extracellular CISs are released into the extracellular milieu, and the released CIS particles attach to the surface of the target cells and inject effectors by puncturing the cell envelope (16, 17). Cryo-electron microscopy (cryo-EM) structural analysis of CISs has supported these mechanistic models. Overall structures of Afp and *Photorhabdus* virulence cassette (PVC) that highly resemble contractile phage tails and potential tail fiber components, which mediate attachment to the target cell, have been identified (16, 17). On the other hand, the previous finding of T6SS*^iv^* showing intracellular localization has suggested that CIS-related nanostructures evolved from a common ancestor into at least two distinct groups that show either extracellular or intracellular localization (Fig. 1) (18). Intracellular localization of SLPs is inferred from the current study together with recent works concerning CIS-like nanostructures of S. coelicolor (41, 42), and this is suggestive of their evolutionary relationship with T6SS*^iv^* (Fig. 1; see Fig. S1 at https://doi.org/10.6084/m9.figshare.22223797). However, there are critical differences in the organization of their gene clusters: genes encoding a tape measure protein and a PAAR domain-containing protein are absent in the SLP gene cluster (Fig. 1A). PAAR domain-containing proteins are needle-like proteins that often contain effector domains and form a spike complex with VgrG protein in both CISs and T6SSs (43). Lack of PAAR domain-containing proteins severely impairs T6SS activity (43). In some cases, effector domains of PAAR domain-containing proteins can be found in VgrG proteins as additional C-terminal domains, for example, in VgrG2b of Pseudomonas aeruginosa T6SS (44). As PAAR domain-containing proteins act as needle tips in known CISs and T6SSs, the absence of both PAAR domain-containing proteins and an additional C-terminal region of VgrG-like protein (Slp5) in the SLP gene cluster suggests a fundamental difference in biological functions between SLPs and other known phage tail-like nanostructures encompassing CISs and T6SSs. Based on these observations and our data, we here propose that SLPs represent a new class of phage tail-like nanostructures that are produced by Gram-positive bacteria and are distinct from typical Gram-negative bacterial CISs and T6SSs.

In Streptomyces coelicolor A3(2), SigE activity is regulated at the level of *sigE* transcription, which is different from the anti-sigma factor-mediated regulation of SigE expressed in other model organisms such as E. coli (45). Antibiotics such as vancomycin and bacitracin are among the known factors that induce *sigE* transcription in *Streptomyces*, thereby targeting the peptidoglycan synthesis pathway (46). This has led to the hypothesis that an intermediate of peptidoglycan biosynthesis or degradation may be the inducing factor of *sigE* transcription (46). In this view, one of the roles of the *Streptomyces* SigE regulon which encompasses penicillin-binding proteins and transpeptidases may be to maintain cell envelope integrity of mycelia by sensing peptidoglycan defects that could be crucial in filamentous bacteria with an apically extended cell shape. The filamentous bacterium *Streptomyces* grows by tip extension but not by lateral growth, which is often observed in unicellular bacteria (26). While lateral cell wall synthesis is not essential for mycelial extension of *Streptomyces*, the lateral cell walls of growing *Streptomyces* mycelia undergo peptidoglycan synthesis (25, 33). Given that the deletion of proteins mediating proper lateral cell wall synthesis, including MreB, in unicellular bacteria markedly decreases resistance to the cell envelope stress of the cells (28), the high sensitivity of S. lividans mutants deficient in either MreB or SLP to certain cell envelope stress suggests that these proteins may support cell envelope integrity and ultimately contribute to phenotypes under the stress conditions.

Our pulldown assays and quantitative proteome analysis indicate the potential association of SLP with 30S ribosomal proteins, which is probably mediated by an interaction between SlpS and ribosomal protein S16 (Fig. 5). Furthermore, our *in vitro* cross-linking experiment suggests EF-Tu1 and EF-G1 as candidates for indirect interaction partners of SLP (Fig. 4). Since it is generally expected that the abundance of EF-G is rather lower than that of EF-Tu in bacterial cells, capturing both of these proteins in a nonspecific manner would be unlikely. In addition, the lack of factors required for stabilization of a dynamic and transient interaction between EF-Tu1/EF-G1 and ribosomal proteins in the buffer would account for the absence of these elongation factors in the list of proteins enriched in Slp5-His_6_ eluates (Fig. 5B). Our results collectively imply that the 30S subunit of the ribosome and SLP interact via protein-protein interactions between ribosomal protein S16 and SlpS, thereby bringing the Slp5 spike complex and EF-Tu1/EF-G1 into spatial proximity at the 30S subunit. As this interaction between SLP and ribosomal proteins would be critically affected by the conformational change of SlpS upon sheath contraction of SLP (Fig. 1B), it can be speculated that the CIS-like contraction mechanism of SLP may dynamically alter the mode of the SLP-ribosome interaction. In addition, it should be noted that MurG, a cytoplasmic cell wall-synthesizing enzyme, and MreB were also enriched in the Slp5-His_6_ eluates (Fig. 5B). A relatively moderate abundance ratio of these proteins would indicate that they interact with the other proteins enriched in the Slp5-His_6_ eluates rather than Slp5-His_6_ itself. SLP could form a multiprotein complex with these cell wall-related proteins, the ribosomal proteins, and other ribosome-associated proteins, which may provide an explanation for the correlation of SLP and 30S ribosomal subunit localization with cell wall synthesis. The putative, complex interaction network involving ribosomal proteins and SLPs might contribute to the hyperosmotic stress resistance of S. lividans by directing the processes of cell envelope maintenance, including synthesis/trafficking of cell wall materials and proteins at the specific region of mycelia. Further analysis of the protein-protein interaction network surrounding SLP within S. lividans mycelia is needed to clarify the detailed molecular mechanism. Additionally, the absence of DivIVA and Scy in the list of the enriched proteins (Fig. 5B) also suggests that SLP proteins and these proteins do not interact directly, although they are partially colocalized within mycelia at the late stage of vegetative growth (Fig. 3A).

Our study also suggests the effects of SLP loss on the susceptibility of S. lividans to certain cell envelope stresses, providing an explanation for studies showing that the colonies of SLP-deficient mutants of S. lividans were severely invaded by fungi at the colony interfaces (12). In these structured microbial communities, where different microorganisms are mixed and compete for niche spaces and nutrients, bacteria experience various stressors. One of these stressors could be osmotic stress created by high local concentrations of extracellular matrices (47). As SLPs are unlikely to exhibit antifungal activity (12), it is possible that SLPs confer resistance to S. lividans mycelia against the osmotic stress that occurs during microbial competition, and this may ultimately prevent the fungal cells from proliferating and invading S. lividans colonies (12). This may increase the survival of S. lividans and improve its ability to acquire niches in densely populated microbial communities in natural environments such as soil and consequently allow the maintenance of SLP genes in the genome. In addition, intracellular localization of SLPs and the relatively simple organization of the SLP genes encoding baseplate complex-related proteins (Fig. 1A) hint at how phages and the related nanomachines have coevolved with their hosts through the loss and/or acquisition of genes.

In summary, we have shown that SLP is associated with the cytoplasmic components of S. lividans and that its loss affects the susceptibility of this bacterium to certain cell envelope stresses. Our findings highlight unique features of a new type of intracellularly localized phage tail-like nanostructures and provide new clues for understanding the functionalities of SLP and the related nanomachines that are highly conserved among *Streptomyces* species.

## MATERIALS AND METHODS

### Strains and culture conditions.

The strains used in this study are listed in Table S1 at https://doi.org/10.6084/m9.figshare.22223797. S. lividans strains were routinely cultured in Bennett's glucose (BeG) medium comprising 0.1% (wt/vol) yeast extract, 0.1% (wt/vol) meat extract, 0.2% (wt/vol) N-Z-Amine, and 1% (wt/vol) glucose. The pH was adjusted to 7.2. For microscopy analysis of substrate mycelia, yeast extract with supplements (YES) medium comprising 0.5% (wt/vol) yeast extract and 1% glucose (wt/vol) was also used for S. lividans culture. For measurement of the growth curve, 10^5^ viable spores were inoculated into 100 mL of liquid BeG medium and incubated at 30°C with shaking at 180 rpm. For phenotypic analysis of the S. lividans colony, S. lividans strains were grown on MS medium comprising 2% (wt/vol) soy flour, 2% mannitol, and 2% (wt/vol) agar. To create a high-osmolarity environment in the medium, 10% (wt/vol) sucrose was added. For measurement of the growth curve and quantification of extracellular DNA, S. lividans strains were grown in 100 mL liquid NMMP medium comprising 0.5% (wt/vol) glucose, 0.2% (wt/vol) ammonium sulfate, 0.5% (wt/vol) Casamino Acids, 0.06% MgSO_4_·7H_2_O, 5% (wt/vol) polyethylene glycol (PEG) 6000, 15 mM potassium phosphate buffer (pH 6.8), 0.0001% (wt/vol) ZnSO_4_·7H_2_O, 0.0001% (wt/vol) FeSO_4_·7H_2_O, 0.0001% (wt/vol) MnCl_2_·4H_2_O, and 0.0001% (wt/vol) CaCl_2_. To create a high-osmolarity environment in the medium, 10% (wt/vol) sucrose was added. For preculture for measurement of the growth curves in Fig. 2E and Fig. S9 at https://doi.org/10.6084/m9.figshare.22223797, we used ISP2 medium comprising 0.4% (wt/vol) yeast extract, 1% (wt/vol) malt extract, and 0.4% (wt/vol) glucose. After 2 days of preculture, mycelia were washed with NMMP medium, gently homogenized, and inoculated into fresh NMMP medium.

### Genetic manipulations.

All primers and plasmids used in this study are listed in Table S2 at https://doi.org/10.6084/m9.figshare.22223797. Nucleotide sequences of genes encoding msfGFP and mScarletI were codon optimized to S. lividans. Gene deletion of S. lividans was performed as follows. The pK18mob plasmid was used as a conjugation vector; it was digested with EcoRI and HindIII and then fused with the amplified flanking regions (approximately 2 kbp each) of the genes of interest by using In-Fusion (Clontech Laboratories, Inc., CA, USA). The constructed plasmid (pK18mob_*sigE*_Del) was transformed into E. coli S17-1. The plasmid was then transferred from the transformed E. coli S17-1 to S. lividans by conjugation. Gene deletion mutants were obtained through homologous recombination events and consequent in-frame deletion of the targeted gene. For *mreB* disruption, the internal sequence of *mreB* was cloned and the resultant plasmid (pK18mob_*mreB*_Del) was transferred to S. lividans. Disruption of *mreB* was completed through a single homologous recombination event. Kanamycin (20 μg/mL) was used as a selection marker. The kanamycin-resistant control strain and the *mreB-msfgfp* strain were constructed using a derivative of integrative pTYM19t plasmid harboring a kanamycin resistance gene instead of the *tsr* gene. *mreB* was complemented by integration of the above-mentioned pTYM19t derivative (48) harboring *mreB* and its promoters at KpnI and HindIII restriction sites (pTYM_*mreB*). *mreB* and its promoters were amplified using primer set *mreB*_12. For fusion of *scy* and *mreB* with msfGFP, the N- or C-terminal regions of *scy* and *mreB* were amplified (primer sets *msfgfp-scy*_12, *msfgfp-scy*_34, *mreB-msfgfp*_12, and *mreB-msfgfp*_34) and fused with a flexible linker and codon-optimized *msfgfp*. The fused sequence was then introduced into the pK18mob plasmid, and each of the resultant plasmids (pK18mob_*msfgfp-scy* and pK18mob_*mreB-msfgfp*) was transferred to S. lividans as described above. Replacement of native *scy* and *mreB* with *msfgfp-scy* and *mreB-msfgfp*, respectively, was completed through homologous recombination events. For fusion of ribosomal protein S6 with mScarletI, the C-terminal region of *rpsF* was amplified (primer set *rpsF-mscarletI*_12 and *rpsF-mscarletI*_34) and fused with a flexible linker and codon-optimized *mscarletI*. This fusion gene was further fused with pK18mob plasmid. The resultant pK18mob_*rpsF-mscarletI* was used for replacement of *rpsF* with *rpsF-mscarletI* at the native chromosomal locus as described above. S. lividans expressing DivIVA-msfGFP was constructed based on the methods described previously (31). In brief, the *divIVA* gene and the flanking regions were cloned, and the gene was fused with *msfgfp* at their C-terminal regions. These sequences were fused to pK18mob, and the resultant plasmid (pK18mob_*divIVA-msfgfp*) was transformed into S. lividans as described above. The *msfgfp*-fused genes were introduced through a homologous recombination event which inserted the *msfgfp*-fused genes with their native promoters into the adjacent noncoding region of the intact genes. This allowed the intact proteins and the labeled proteins to be simultaneously expressed and could minimize the effects of msfGFP fusion to the target proteins on the morphologies of the S. lividans mycelia.

For overexpression of SLP proteins, pGMH plasmids were constructed as follows. The backbone region containing the replication origin and antibiotic resistance genes of pGM1192 plasmid was amplified by inverse PCR (primer set pGM1192_Inverse_1 and _2). The resulting fragment was fused with *hrdB* promoter, and each of SLP genes was cloned from the S. lividans genome (primer sets P*_hrdB_*_1 and _2, *slp5*_pGMH_1 and _2, *slp7*_pGMH_1 and _2, and *slp8*_pGMH_1 and 2) by In-Fusion. The resulting plasmid was amplified in E. coli strains DH5α and S17-1 in the presence of apramycin. After conjugation, the transformants of S. lividans were selected by using thiostrepton.

For overexpression of His_6_-Slp5, the *slp5* gene cloned from the S. lividans genome (primer set *Hisslp5*_PET15b_1 and _2) was fused with pET15b digested with NdeI and BamHI by In-Fusion. The resulting plasmid was introduced into E. coli BL21(DE3).

For the coelution assay of ribosomal protein S16 and SlpS, we constructed the following plasmids for heterologous expression of the recombinant proteins in E. coli: pET51b::*streptagII-rpsP-his_10_* and pET26b::*slpS-his_6_*. *rpsP* and *slpS* encoding ribosomal protein S16 and SlpS, respectively, were cloned from the S. lividans genome using the primer set *slpS*_pET26b_12 and *rpsP*_pET51b_12. *rpsP* was fused with pET51b(+) digested with BamHI and HindIII by In-Fusion. *slpS* was fused with pET26b(+) digested with NdeI and BamHI by In-Fusion. The resulting plasmids were introduced into E. coli BL21(DE3).

### Extraction of SLPs.

SLPs were extracted from *Streptomyces* mycelia as follows. Viable spores (10^5^ per plate) were spread onto cellophane placed on a BeG agar plate. After incubation at 30°C for 3 to 5 days, the mycelia were scraped off the plate and resuspended in lysis buffer containing 50 mM Tris-HCl buffer (pH 7.5), 150 mM NaCl, 1 mg/mL lysozyme, 100 μg/mL DNase, 1% (vol/vol) Triton X-100, and a protease inhibitor cocktail. The resuspended solution was incubated at 37°C for 2 h. After centrifugation at 15,000 × *g* for 10 min, the supernatant was ultracentrifuged at 200,000 × *g* for 60 min. The pellets were resuspended in resuspension buffer containing 50 mM Tris-HCl buffer (pH 7.5), 150 mM NaCl, and a protease inhibitor cocktail. The resultant solutions were subjected to further analysis. For TEM, the extracted SLPs were attached to thin carbon film-coated TEM grids (Alliance Biosystems, Osaka, Japan) and washed with H_2_O. SLPs were then visualized by negative staining.

### Microscopic analysis.

CLSM was performed using an LSM780 confocal microscope equipped with an EC Plan-Neofluar 100×/1.3 oil Ph3 objective (Carl Zeiss, Oberkochen, Germany), an Olympus FV1200 microscope (Olympus, Tokyo, Japan) equipped with a UPlanApo 100 × 1.40 numerical aperture (NA) oil-immersion lens (Olympus), and an Olympus SpinSR10 microscope (Olympus) equipped with an UPLAPO OHR 60 × 1.5 NA (Olympus) or UAPON OTIRF 100 × 1.49 NA (Olympus) objective. Colocalization analysis was performed using Fiji and a Coloc2 plugin.

The mycelia of S. lividans were visualized as follows. For localization analysis of the fusion proteins, S. lividans spores were inoculated into 4 mL of liquid YES medium and incubated at 30°C for 20 to 24 h. This preculture was further inoculated into 1 mL of fresh YES liquid medium and incubated at 30°C for 20 to 24 h. For live cell imaging (Fig. 1D; see Movie S1 in the supplemental material), the spores (10^2^ viable spores) were inoculated onto cellophane (approximately 4 cm^2^) placed on solid BeG medium and then allowed to dry. The cellophane and the solid medium were then inverted and placed on a glass-bottom dish. The dish was precultured at 30°C for 48 h and then subjected to microscopy analysis on an Olympus SpinSR10 microscope. During observation, the temperature was kept at 30°C. For observation of Van-Fl spots and measurement of branching frequency, S. lividans mycelia were grown in a BeG slide culture at 30°C for 3 days (late stage of vegetative growth).

The colony was observed using an Axio Zoom microscope (Carl Zeiss) equipped with a PlanApo Z 0.5× objective and an AxioCam MR R3 microscope camera. For time-lapse imaging of colony growth, the plate was held between thermoplates and kept at approximately 30°C.

HADA, a synthetic d-amino acid with a fluorescent moiety, was incorporated into peptidoglycan of S. lividans as follows. S. lividans mycelia at late exponential growth phase were cultivated in liquid YES medium containing 20 μg/mL HADA at 30°C for 30 min and then washed with fresh YES medium. The labeled mycelia were immediately subjected to CLSM analysis.

SEM analysis was performed as follows. S. lividans TK23 and Δ*slpS* spores were inoculated onto MS medium supplemented with 10% (wt/vol) sucrose and incubated at 30°C for 7 days. The colonies were scraped off and fixed with 2.5% (vol/vol) glutaraldehyde for 2.5 h. The fixed colonies were washed with 0.1 M phosphate buffer (pH 7.4) three times. The colonies were further treated with 1% (wt/vol) OsO_4_ and then dehydrated sequentially with 50%, 70%, 90%, and 100% ethanol. The dehydrated samples were lyophilized and subjected to SEM analysis.

For TEM, samples were attached to thin carbon film-coated TEM grids (Alliance Biosystems) and washed with H_2_O. The samples were then visualized by negative staining.

### Western blot analysis.

Rabbit anti-SlpS antiserum, anti-Slp5 antiserum, and anti-RpoD antiserum specific for the internal peptides (15 amino acids each) of the corresponding proteins were prepared at Eurofins Genomics (Tokyo, Japan). Proteins were separated by SDS-PAGE and then electroblotted onto polyvinylidene difluoride (PVDF) membranes. After blocking with 5% (wt/vol) skim milk, the blots were incubated with anti-SlpS antiserum, anti-Slp5 antiserum, anti-RpoD antiserum, anti-His_6_ antibody AB9108 (Abcam, Cambridge, UK; diluted to 0.02%), or anti-GFP antibody AB290 (Abcam; diluted to 0.02%) at room temperature for 60 min and with horseradish peroxidase (HRP)-conjugated secondary antibodies at room temperature for 60 min. The immunoreactive proteins were detected with Pierce ECL Plus Western blotting substrate (Thermo Fisher) or ImmunoStar LD (FUJIFILM Wako Chemicals, Osaka, Japan). For detection of the biotin-labeled proteins, the electroblotted proteins were reacted with streptavidin-HRP conjugate (Tokyo Chemical Industry Co., Ltd., Tokyo, Japan).

### Isolation of membrane fractions.

Membrane fractions were isolated as follows. S. lividans mycelia were grown in 4 mL liquid YES medium for 2 days with shaking. Mycelia were then collected by centrifugation and resuspended in 1 mL PBS. The mycelia were disrupted by sonication. The whole mycelial extracts were diluted with 5 mL of 60% (wt/vol) iodixanol. A 400-μL volume of this mixture was placed at the base of a 4-mL tube, and then 400 μL of 45, 40, 35, 30, 25, 20, and 15% iodixanol solutions were layered on top of the suspended solution to make a density gradient in the tube. The tube was immediately ultracentrifuged for 3 h at 100,000 × *g* at 4°C. After ultracentrifugation, fractions were collected and subjected to Western blot analysis.

### Affinity-based coelution assay for ribosomal protein S16 and SlpS.

E. coli harboring pET51b::*streptagII-rpsP-his_10_* and pET26b::*slpS-his_6_* (see “Genetic manipulations”) were cultivated in 4 mL LB medium containing the appropriate antibiotics. In the case of pET51b::*streptagII-rpsP-his_10_*, the medium was supplemented with 0.5% (wt/vol) glucose to suppress the basal expression of *rpsP*. Subsequently, 1 mL of the preculture was transferred to a fresh 100-mL volume of LB medium containing 50 μg/mL kanamycin or 50 μg/mL ampicillin. After cultivation at 37°C for 90 min, the cultures were immediately cooled on ice. After cooling, protein expression was induced by adding 0.1 mM isopropyl β-d-1-thiogalactopyranoside (IPTG) to the medium and cultivating the cells at 18°C for 16 h in the presence of the inducer. Cells were harvested by centrifugation of 200 mL of the culture and then resuspended in 4 mL of 20 mM Tris-HCl buffer (pH 8.0), followed by cell lysis by sonication. After removal of debris by centrifugation, the lysates were loaded onto a His GraviTrap column, 1 mL (Cytiva), equilibrated with 20 mM Tris-HCl buffer (pH 8.0) containing 10 mM imidazole. After washing with the same buffer, proteins were eluted from the column with 20 mM Tris-HCl buffer (pH 8.0) containing 500 mM imidazole. The buffer was replaced with 20 mM HEPES-NaOH buffer (pH 7.4) containing 5 mM MgCl_2_, 150 mM NaCl, and 10% glycerol (vol/vol) for the following coelution assay. The partially purified StrepTagII-protein S16-His_10_ and SlpS-His_6_ were mixed to final concentrations of 0.2 mg/mL and 0.1 mg/mL, respectively. The solutions containing each of the proteins were prepared as controls. These protein solutions were incubated at room temperature for 15 min and then loaded onto a Capturem streptavidin miniprep column (Clontech). After washing twice with PBS, the captured proteins were eluted with 0.1 M glycine (pH 2.5). The eluted proteins were separated by SDS-PAGE and detected by Western blotting with anti-His_6_ antibody and streptavidin-HRP conjugate (see “Western blot analysis”).

### Quantification of extracellular DNA.

S. lividans strains were grown as described in “Strains and culture conditions” and removed by centrifugation (7,000 × *g*, 10 min). Double-stranded DNA in the resulting supernatant was then quantified using PicoGreen (Life Technologies, Carlsbad, CA, USA). The DNA concentration was calculated based on the standard curve obtained from the standard DNA.

### Bioinformatic analysis.

The amino acid sequences of the CIS tube proteins were obtained from a database for eCIS (3). Evolutionary analyses were conducted in MEGA X (49). The tree was drawn to scale, with branch lengths in the same units of the evolutionary distances used to infer the phylogenetic tree. All ambiguous positions were removed for each sequence pair (pairwise deletion option). Bootstrap values were calculated from 500 replicates.

### Cross-linking, affinity-based purification, and mass spectrometry analysis of interaction partners of Slp5.

E. coli harboring pET15b::*his_6_-slp5* (see “Genetic manipulations”) was precultured in 4 mL LB containing ampicillin. One milliliter of the preculture was transferred to a fresh 200 mL LB medium containing 50 μg/mL ampicillin. After cultivation at 37°C for 90 min, the cultures were cooled at room temperature and then supplemented with 0.1 mM IPTG. After cultivation at 18°C for 16 h, the cells were harvested by centrifugation, suspended with 20 mM HEPES-NaOH (pH 8.0), and disrupted by sonication. After removal of debris, the resulting cell lysates were loaded into AKTA purifier systems (GE Healthcare) equipped with a HisTrap 5-mL column (GE Healthcare). The purification conditions were as follows: buffer A, 10 mM HEPES-NaOH (pH 8.0); buffer B, 10 mM HEPES-NaOH (pH 8.0) and 500 mM imidazole; flow rate, 1 mL/min; column wash, 2 column volumes with 4% buffer B; fraction volume, 3 mL; temperature, 4°C; linear gradient after washing, 10 column volumes with 4 to 100% buffer B. The fractions with no visible contaminating band were obtained, and the buffer was replaced with 10 mM HEPES-NaOH (pH 7.4) using an Amicon Ultra-4 centrifugal filter unit (Millipore). Note that imidazole was almost completely removed from the buffer. We confirmed that Slp5 was purified by Western blot analysis using anti-Slp5 antiserum.

Prior to the cross-linking experiment, S. lividans TK23 was cultivated in 100 mL YES liquid medium for 24 h. The mycelia were harvested by centrifugation and resuspended with 1 mL PBS. After sonication, debris were removed by filtration (0.45 μm). These mycelial lysates were used immediately for cross-linking as described below.

The following procedures were conducted in the dark. A 1.1-mg amount of sulfo-SBED (Thermo Fisher) was dissolved in 25 μL dimethyl sulfoxide. This solution was added to 600 μL of 10 mM HEPES-NaOH (pH 7.4), and then 200 μL of Slp5 (4 mg/mL) or BSA (5 mg/mL in 10 mM HEPES-NaOH [pH 7.4]) was added immediately to the solution. These mixtures were kept on ice for 2 h to label surface-exposed amino groups with the cross-linking reagent. After the reaction, these solutions were dialyzed twice with the same buffer at 4°C for 16 h. We confirmed that the proteins were not precipitated during these procedures. A 250-μL volume of the dialyzed solution was mixed with the same volume of the S. lividans lysates. After incubation at room temperature for 5 min, the cross-linking reaction was provoked by UV light at 365 nm. 2-Mercaptoethanol was then added to the reaction mixtures at a final concentration of 100 mM to cleave the S-S bond in the cross-linking reagent. The mixtures were dialyzed with 50 mM sodium phosphate buffer (pH 7.0) containing 1 mM EDTA and 1 M NaCl.

The potential interaction partners were isolated and identified as follows. The above-described protein solutions were loaded into a Capturem streptavidin miniprep column. After washing with PBS, the proteins captured by the columns were eluted with 0.1 M glycine (pH 2.5). The eluted proteins were separated by SDS-PAGE and analyzed by Western blotting (see “Western blot analysis”). The bands visualized with CBB were excised from the gel and subjected to peptide mass fingerprinting as follows. The isolated proteins were digested with trypsin, and then the carbamidomethyl group was added to the -SH group of cysteine residues of the peptides. The resulting peptides were analyzed by matrix-assisted laser desorption ionization–time of flight (MALDI-TOF) mass spectrometry. The obtained mass spectra derived from these peptides were analyzed by a MASCOT database search (Matrix Science Ltd., London, UK). Proteins that showed the highest scores (above the significance threshold) were assigned to the bands.

### Proteomic analysis.

Spores of S. lividans strains harboring pGMH::*slp5* or pGMH::*slp5-his_6_* were inoculated into liquid BeG medium and grown for 2 days with shaking. After cultivation, mycelia were collected by centrifugation, washed with 20 mM Tris-HCl (pH 8.0), and then disrupted by sonication. The lysates were filtered with a 0.45-μm filter. The filtered lysates were then loaded into a His GraviTrap column, 1 mL (Cytiva). After the column was washed with 10 mL of 10 mM Tris-HCl (pH 8.0) supplemented with 20 mM imidazole, proteins were eluted from the column with 3 mL of elution buffer containing 10 mM Tris-HCl and 500 mM imidazole (pH 8.0). The buffer was replaced with 10 mM HEPES-NaOH (pH 7.5) using an Amicon Ultra-4, and the concentrated proteins were subjected to proteomic analysis as described below.

Proteins were separated by SDS-PAGE and treated by in-gel digestion with trypsin. The digested samples were purified by using ZipTips and were analyzed by advance nanoflow ultrahigh-performance liquid chromatography (UHPLC) (Bruker, MA, USA) on a Q Exactive quadrupole orbitrap mass spectrometer (Thermo Fisher, MA, USA) equipped with a Zaplous column (0.2 inside diameter [i.d.] by 50 mm; AMR, Inc., Japan, Tokyo) under the following conditions: column temperature, 35°C; mobile phase, gradient mixture of solvent A (0.1% formic acid) and solvent B (acetonitrile); flow rate, 1.5 mL/min; and gradient elution, 0 min (solvent A:solvent B = 95:5), 20 min (35:65), and 21 min (5:95). For protein identification, quantification, and comparison between two groups, a database search was performed by a label-free quantification workflow in Proteome Discoverer 2.5 inserted with a Sequest HT search engine with percolator against the genome of S. lividans. Abundances of peptide spectral matches (PSMs) were averaged from the three technical replicates. Abundance ratios and *q* values were calculated from the results for three biological replicates.

### Data availability.

The data sets supporting the current study are available from the corresponding authors on request. See also the data at https://doi.org/10.6084/m9.figshare.22223797.
